# Buprenorphine and naloxone access in pharmacies within high overdose areas of Los Angeles during the COVID-19 pandemic

**DOI:** 10.1186/s12954-022-00651-3

**Published:** 2022-06-29

**Authors:** David Dadiomov, Maria Bolshakova, Melania Mikhaeilyan, Rebecca Trotzky-Sirr

**Affiliations:** University of Southern California School of Pharmacy, 1985 Zonal Ave, Los Angeles, CACA 90089 USA

**Keywords:** Pharmacy, Naloxone, Buprenorphine, Opioid, Overdose, Availability, Urban

## Abstract

**Background:**

Buprenorphine and naloxone are first-line medications for people who use opioids (PWUO). Buprenorphine can reduce opioid use and cravings, help withdrawal symptoms, and reduce risk of opioid overdose. Naloxone is a life-saving medication that can be administered to reverse an opioid overdose. Despite the utility of these medications, PWUO face barriers to access these medications. Downtown Los Angeles has high rates, and number, of opioid overdoses which could potentially be reduced by increasing distribution of naloxone and buprenorphine. This study aimed to determine the accessibility of these medications in a major urban city by surveying community pharmacies regarding availability of buprenorphine and naloxone, and ability to dispense naloxone without a prescription.

**Methods:**

Pharmacies were identified in the Los Angeles downtown area by internet search and consultation with clinicians. Phone calls were made to pharmacies at two separate time points–September 2020 and March 2021 to ask about availability of buprenorphine and naloxone. Results were collected and analyzed to determine percentage of pharmacies that had buprenorphine and/or naloxone in stock, and were able to dispense naloxone without a prescription.

**Results:**

Out of the 14 pharmacies identified in the downtown LA zip codes, 13 (92.9%) were able to be reached at either time point. The zip code with one of the highest rates of opioid-related overdose deaths did not have any pharmacies in the area. Most of the pharmacies were chain stores (69.2%). Eight of the 13 (61.5%) pharmacies were stocked and prepared to dispense buprenorphine upon receiving a prescription, and an equivalent number was prepared to dispense naloxone upon patient request, even without a naloxone prescription. All of the independent pharmacies did not have either buprenorphine or naloxone available.

**Conclusions:**

There is a large gap in care for pharmacies in high overdose urban zip codes to provide access to medications for PWUO. Unavailability of medication at the pharmacy-level may impede PWUO ability to start or maintain pharmacotherapy treatment. Pharmacies should be incentivized to stock buprenorphine and naloxone and encourage training of pharmacists in harm reduction practices for people who use opioids.

## Introduction

Opioid use poses significant risks to public health through overdose deaths [1]. Naloxone and buprenorphine are two common medications used as harm reduction strategies for people who use opioids (PWUO) that can reduce overdose mortality and secondary opioid-related outcomes [2–4]. Naloxone is an opioid antagonist that can counter the effects of opioid overdose [4] and is commonly administered intranasally (Narcan formulation) when an opioid overdose is suspected. Increasing distribution of naloxone to laypersons likely to witness or experience an overdose and first-responders may help reduce overdose deaths by 21% [5]. Buprenorphine is a partial opioid agonist and is commonly prescribed under the name Suboxone®, which is a buprenorphine + naloxone combination. There is strong evidence to support the use of opioid agonists and partial agonists such as buprenorphine to reduce opioid use and retain patients in treatment, and can be given as a stand-alone treatment, or as an effective addition to other forms of counseling and addiction support groups [3]. It has also been shown to reduce opioid cravings, reduce risk of overdose, and helps with withdrawal symptoms [6, 7].

All 50 US states and the District of Columbia have some form of naloxone access laws which help widen the access and distribution of naloxone, although the laws vary significantly by state [8]. Most US states allow for a ‘standing order’ or third-party prescription which gives pharmacists permission to issue a prescription for individuals at risk for overdose. In some states such as California, pharmacists are allowed to dispense naloxone to any individual, regardless of overdose risk, without a prescription. Pharmacies may choose not to stock naloxone for various reasons. Pharmacists in some states must have specific training on naloxone as well as providing education on administration of the drug to the recipient which may be time-consuming and costly [9]. Pharmacists may also have stigma towards people who use drugs, may be concerned with liability issues, lack the confidence to dispense naloxone and communicate with patients, be misinformed about legal and pharmacological aspects of naloxone, and have inadequate staffing and time to ensure proper naloxone distribution protocol [9–11].

California had the largest number of drug-related deaths in the country in 2019 [12], and some of the highest rates occur in densely populated urban areas, such as Downtown Los Angeles. Amidst the COVID-19 pandemic, Los Angeles County has seen a 52% increase in drug overdose deaths [13] higher than the national increase of 38% during the pandemic [14]. Nevertheless, two years after the implementation of legislation allowing pharmacists to furnish naloxone to individuals without a prescription, less than a quarter (23.5%) of California retail pharmacies were able to dispense naloxone to patients without a physician prescription, and only 50% of these pharmacies had nasal naloxone in stock [11].

The disparity between high overdose rates and pharmacy access to buprenorphine has been reported nationwide [15]. Despite an increasing number of prescribers of buprenorphine [16] and increased access to buprenorphine initiation due to expansion of telemedicine during the COVID-19 pandemic [17], prescriptions of buprenorphine for new patients decreased during the beginning of the COVID-19 pandemic [18]. Out of pharmacies that provided naloxone without a prescription in California, only 5% also offered buprenorphine-naloxone tablets to their patients [11]. It appears that while legislation is breaking down certain barriers to harm reduction medications, access to these drugs may still be hindered by lack of availability at pharmacies. The readiness for pharmacies to stock and dispense buprenorphine and naloxone in major urban areas has been under-studied. Thus, we aimed to regionally examine access to buprenorphine and naloxone in community pharmacies in a major urban city with high opioid overdose rates (Los Angeles, California).

## Methods

**Fig. 1:**
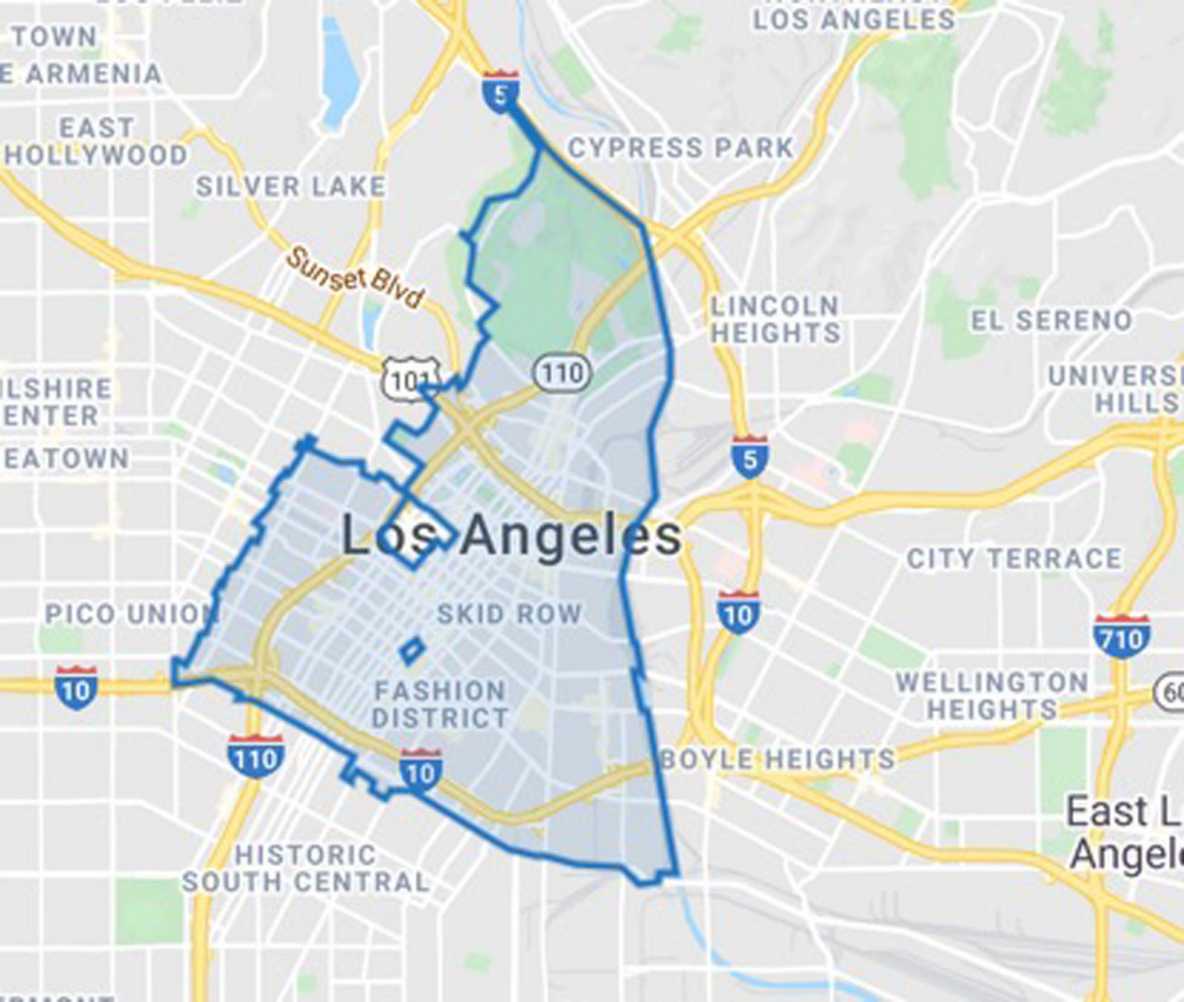
Map of zip codes searched for pharmacies

We conducted a descriptive study during the COVID-19 pandemic assessing the availability of buprenorphine and naloxone in community pharmacies located within the Downtown Los Angeles zip codes of high overdose risk [19]. We excluded zip codes with a population of less than 1,000. Searches were conducted using Google Maps, Yelp, and input from clinicians. From September 2020 to March 2021, we conducted phone calls to both chain and independent pharmacies within the zip codes: 90,012, 90,013, 90,014, 90,015, 90,017, and 90,021 (Fig. 1).^1^ The University of Southern California Institutional Review Board granted this study as “Exempt”.

Initial calls were made by graduate researchers (MB and MM) in September 2020 during business hours and followed a semi-structured script. Each call examined whether the pharmacist was willing and able to fill a prescription from a DATA 2000 waivered addiction physician for buprenorphine/naloxone 8 mg/2 mg sublingual films for a patient with opioid use disorder for a 2-week supply. Pharmacists were also asked whether they were able and willing to dispense naloxone without a prescription to patients per California regulations. If out of stock on buprenorphine/naloxone films, the pharmacist was asked whether any other formulations or dosages were available, regardless of generic or brand name. Responses were anonymously documented, and assessment followed up for a second round of calls in March. All results in 2021 were compared to the preceding year.

We also extracted data on opioid-overdose deaths and total population by zip code using census estimates [20] and the California Overdose Surveillance Dashboard [19] to produce the opioid-related overdose death rate per 100,000 residents among surveyed zip codes to contextualize our results within the downtown Los Angeles area.

## Results

We identified and called 14 pharmacies, the majority of which (57%) were chain pharmacies. There were no pharmacies identified through searches in the 90,021 zip code, known as the Wholesale District, which has the second highest age-adjusted rate of opioid-related deaths (317.03) in the downtown Los Angeles area (see footnote for Fig. 1). Three pharmacies were either closed or could not be reached by phone during the first round of calls; however, by the second round of calls, only one pharmacy was unable to be reached, for a total of 13 responding pharmacies (92.9%) over the two rounds of calls. An additional PDF file shows the data we collected over the two rounds of calls, including comments made regarding naloxone availability by pharmacists. 

**Fig. 2:**
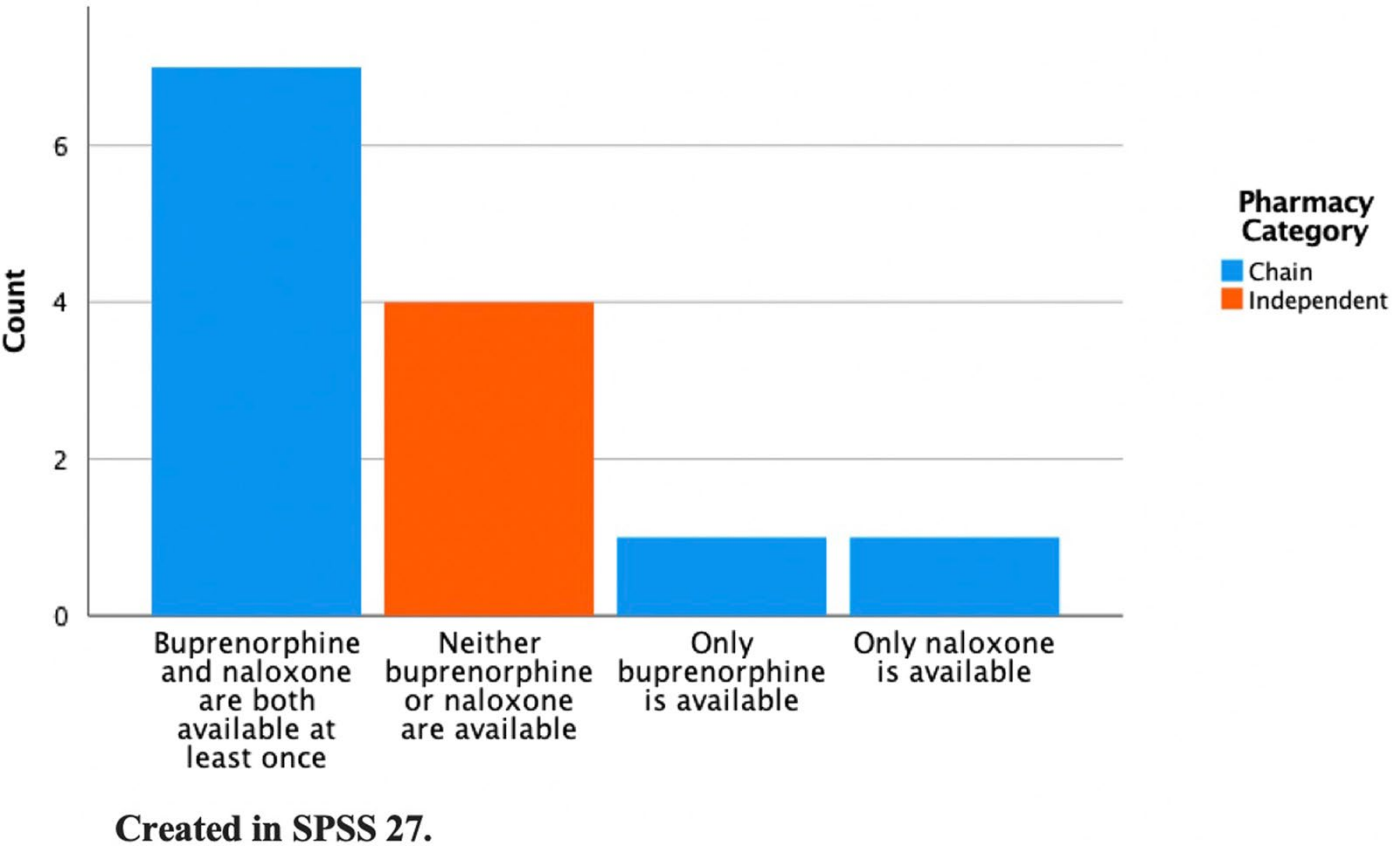
Availability of buprenorphine and naloxone by pharmacy category

Six out of 13 pharmacies had buprenorphine stocked both rounds of calls (46%), and a total of 8/13 (61.5%) of responding pharmacies had buprenorphine in stock either in September 2020 or March 2021 during normal operating hours (Table 1). Over the two rounds of calls, eight pharmacies (61.5%) were able to provide naloxone without a prescription or allowed pharmacists to write the prescription and dispense it, and all 8 (100%) of these pharmacies had the naloxone in stock (Table 1). One pharmacy (7.7%) had buprenorphine but not naloxone, and another had naloxone but no buprenorphine in stock, for a total of 7 (53.8%) pharmacies that had both buprenorphine and naloxone in stock. None of the independent pharmacies had naloxone or buprenorphine in stock. Figure 2 displays availability of buprenorphine and naloxone by pharmacy category (chain or independent).

**Table 1:** Pharmacy Availability of Buprenorphine and Naloxone

Variable	Total number of responding pharmacies = 13 *N* (%)
Pharmacy type	
Chain	9 (69.2%)
Independent	4 (30.8%)
Buprenorphine in stock both rounds	6 (46.2%)
Both buprenorphine and naloxone are available at least once	7 (53.8%)
Buprenorphine in stock at least once	8 (61.5%)
Naloxone in stock at least once	8 (61.5%)

## Discussion

This study demonstrates that buprenorphine for treatment of opioid use disorder is not universally available in a highly populous urban area with high rates of opioid overdose, nor is naloxone for opioid overdose reversal. The opioid-related overdose death rate per 100,000 residents averages 144.93 (ranging from 11.66 to 317.03) among the combined zip codes of pharmacies that we surveyed. There was not a single pharmacy in the zip code with the second highest overdose rate in the county (90,021) [19]. Collectively, these pharmacies practice within zip codes serving 111,118 people [20].

Patients already face multiple internal and structural barriers to buprenorphine initiation such as stigma, past treatment experiences, logistics, and knowledge of medications [21] such that receiving a prescription for buprenorphine is big step that may have required time, commitment and energy. Many patients that are unable to readily travel to various pharmacies, such as patients experiencing homelessness, are adversely impacted by the lack of availability of buprenorphine and naloxone within high-risk zip codes. Arriving to pick up a harm reduction medication and finding out that it is not currently in stock has led to frustration, confusion, and withdrawal symptoms among patients [22], and could potentially cause a patient to give up on the process altogether.

One limitation of this study is the use of Google Maps and Yelp to identify pharmacies, as some may not be listed online or may have inaccurate information such as phone numbers and addresses. However, we thoroughly searched the identified map perimeter and consulted with addiction medicine clinicians at Los Angeles County Hospital to ensure we were capturing all of the common pharmacies used by patients in the downtown area. We did not include the 90,071 zip code in our search or analysis, which has one independent pharmacy in the area and the highest opioid-related overdose death rate in LA County because of the likely error margin that comes with such a small population. A strength of this study is that it is the first known investigation of availability of buprenorphine and naloxone during the Covid-19 pandemic in a major urban area.

Although California state law allows pharmacies to furnish naloxone without a prescription, about 40% of pharmacies surveyed in our study did not provide this service. Pharmacist barriers to naloxone dispensing include inadequate training in identifying and educating patients at risk of overdose, limited time to educate patients, lack of confidence communicating with patients about naloxone, and uncertainty about naloxone access laws [9–11]. Most of the chain pharmacies in our study had both naloxone or buprenorphine in stock, or at least one of the two, likely due to company-wide policy. Conversely, none of the independent pharmacies had buprenorphine or naloxone available. Our data are congruent with other recent studies that investigated availability of buprenorphine and naloxone in community pharmacies along the same rates (40% for buprenorphine, 60% for naloxone) and corroborate findings that chain pharmacies are more likely to have the medications in stock compared to independent pharmacies [23, 24].

## Conclusions

This study is unique in that we specifically targeted pharmacies in high-overdose zip codes in a major urban area that saw 1,300 opioid-related overdose deaths in 2020 [19]. Our findings combined with similar studies indicate that a large portion of pharmacies in the USA are not providing evidence-based, first-line treatment for a genuine, chronic condition such as opioid use and/or dependence.

A systematic review found that additional pharmacist training was a facilitator to pharmacists feeling comfortable dispensing naloxone and communicating with patients; however, only 19 US states and some specific institutions and multi-chain pharmacies require naloxone training programs for pharmacists [9]. Additionally, education on the positive impact of harm reduction practices such as naloxone and syringe dispensing can help reduce stigma and promote harm reduction service availability [25]. Pharmacies, particularly independent stores, should be encouraged and incentivized to keep buprenorphine and naloxone in stock, and to provide training to their pharmacists on managing patients with opioid use disorder and educating patients on using naloxone. While relaxed legislation of buprenorphine and naloxone may increase access to treatment, pharmacies should ensure equitable access to buprenorphine and naloxone.

## Data Availability

All data generated or analyzed during this study are included as links in the references [CA Dashboard; LA Census] and this article’s additional files.

## Abbreviation

PWUO: People who use opioids

## Footnotes

1 The California Overdose Surveillance data list the zip code 90071 as having the highest opioid-related overdose death age-adjusted rate per 100,000 residents in Los Angeles County. However, according to the 2019 Census estimate, this zip code only has about 126 people (15 in 2010). Thus, we excluded 90071 from the search due to likelihood of skewed data impacting our results.
